# Spectroscopic Study of Plasma Polymerized a-C:H Films Deposited by a Dielectric Barrier Discharge

**DOI:** 10.3390/ma9070594

**Published:** 2016-07-19

**Authors:** Thejaswini Halethimmanahally Chandrashekaraiah, Robert Bogdanowicz, Eckart Rühl, Vladimir Danilov, Jürgen Meichsner, Steffen Thierbach, Rainer Hippler

**Affiliations:** 1 Institut für Physik, Ernst-Moritz-Arndt-Universität Greifswald, Felix-Hausdorff-Str. 6, Greifswald 17489, Germany; tch@pdx.edu (T.H.C.); rbogdan@eti.pg.gda.pl (R.B.); Vl.danilov@gmail.com (V.D.); meichsner@physik.uni-greifswald.de (J.M.); 2 Faculty of Electronics, Telecommunications and Informatics, Gdansk University of Technology, 11/12 G. Narutowicza St., Gdansk 80-233, Poland; 3 Institut für Chemie und Biochemie-Physikalische und Theoretische Chemie, Freie Universität Berlin, Takustr. 3, Berlin 14195, Germany; ruehl@chemie.fu-berlin.de (E.R.); thierbach@chemie.fu-berlin.de (S.T.)

**Keywords:** dielectric barrier discharge (DBD), thin film deposition, a-C:H films, atmospheric pressure, hydrocarbon plasma, 78.20.-e, 78.30.-j, 78.66.Qn, 81.15.Gh

## Abstract

Plasma polymerized a-C:H thin films have been deposited on Si (100) and aluminum coated glass substrates by a dielectric barrier discharge (DBD) operated at medium pressure using C_2_H*_m_*/Ar (*m* = 2, 4, 6) gas mixtures. The deposited films were characterized by Fourier transform infrared reflection absorption spectroscopy (FT-IRRAS), Raman spectroscopy, and ellipsometry. FT-IRRAS revealed the presence of *sp*^3^ and *sp*^2^ C–H stretching and C–H bending vibrations of bonds in the films. The presence of D and G bands was confirmed by Raman spectroscopy. Thin films obtained from C_2_H_4_/Ar and C_2_H_6_/Ar gas mixtures have I_D_/I_G_ ratios of 0.45 and 0.3, respectively. The refractive indices were 2.8 and 3.1 for C_2_H_4_/Ar and C_2_H_6_/Ar films, respectively, at a photon energy of 2 eV.

## 1. Introduction

In recent years, plasma deposited amorphous hydrogenated carbon (a-C:H) films have been widely used in scientific and industrial fields due to their excellent mechanical, chemical, tribological, optical, and electronic [1,2,3,4,5] properties. Hydrogenated carbon films are frequently classified into four classes: polymer-like films with the highest hydrogen content of up to 50% and up to 60% of hydrogen-terminated *sp*^3^ bonds, diamond-like films with an intermediate H content (about 30%) and a large fraction of C–C *sp*^3^ bonds, hydrogenated tetrahedral carbon films (ta-C:H) with a typically hydrogen content of 25% and the highest *sp*^3^ content of about 70%, and graphite-like films with low hydrogen (<20%) but large *sp*^2^ contents [6]. Due to these properties, hydrogenated carbon films can be used for super-hard materials [1], biomaterials [7], wear-resistant coatings [8], optical coatings [9], field enhanced conductors [10], smart polymer and biopolymer films [11,12], and hydrogen storage devices [13]. Film characteristics can be suitably tailored for particular applications employing different deposition techniques and precursor gas mixtures, e.g., chemical vapor deposition (CVD) [14], sputtering [15], plasma enhanced chemical vapor deposition (PECVD) [16], and pulsed laser deposition (PLD) [17].

In the current study, we have used a dielectric barrier discharge (DBD) for the preparation of a-C:H thin films [18,19,20]. The advantage of using DBD is that this technique does not involve physical sputtering resulting in a long life time of the deposition device and that a high electron density (10^11^ cm^−3^) can be achieved during film deposition. DBDs when operated at atmospheric pressure do not require an expensive vacuum system which saves costs and allows for an easy handling of deposited films during and after deposition. A certain disadvantage is the narrow discharge gap of typically 0.1 cm or less [21]. In this work we have employed a DBD at a medium pressure of 300 mbar for deposition of amorphous hydrogenated carbon films. It is well known that the choice of a suitable hydrocarbon precursor can result in different film properties. The use of C_2_H*_m_*/Ar (*m* = 2, 4, 6) gas mixtures in the present work extends previous investigations employing CH_4_ precursors and allows for a tailoring of film properties into the desired direction. A plasma chemical characterization of the employed DBD based on mass spectrometry and infrared spectroscopy has been published recently [22]. The deposited films were characterized by Fourier transform infrared reflection absorption spectroscopy (FT-IRRAS), Raman spectroscopy, and spectroscopic ellipsometry (SE).

## 2. Experimental 

The experimental setup has been described in detail elsewhere [22,23,24,25,26]. Briefly, it consists of a plasma chamber with inner dimensions for height, length, and width of 12.3 cm, 18.0 cm, and 15.0 cm, respectively. The two discharge electrodes, each with a length of 8.3 cm, a width of 3.3 cm, and a thickness of 0.15 cm, are made of stainless steel and embedded in a rectangular shaped poly-methyl methacrylate (PMMA) case. Both electrodes are covered by dielectrics, the upper powered electrode was covered by Al_2_O_3_ (ε ≈ 10) and the lower grounded electrode was covered by glass (ε ≈ 3.8). The two electrodes are separated 2 mm or 1.5 mm by a PTFE spacer. The substrate was placed on the glass electrode for thin film deposition. Two different kinds of substrate materials were used for the deposition experiments: aluminum coated glass substrates for FT-IRRAS analysis with dimensions of 6 × 2 cm^2^ and silicon (100) wafers with dimensions of 2 × 3 cm^2^ for Raman spectroscopy and ellipsometric studies. A highly reflecting metallic coating of about 190 nm thickness on the glass substrate was prepared by thermal evaporation of aluminum under vacuum conditions.

The plasma chamber was pumped by a membrane pump down to about 10 mbar. The pressure was monitored using a mechanical pressure gauge connected to the chamber. The pressure inside the chamber was controlled by two gas flow controllers for the hydrocarbon and argon gases and by a needle valve between the chamber and the membrane pump. For all experiments a hydrocarbon-to-argon ratio of 1:2 was used. The chamber was filled with hydrocarbon and argon gas to a partial pressure of 100 mbar and 200 mbar, respectively, prior to the plasma operation. Pump and gas flow were switched off once the chamber was filled.

The high voltage power supply consists of a frequency generator delivering a sinusoidal output that is fed into an audio amplifier. The amplifier can operate up to 500 W; its output is fed into a spark plug transformer. The experiments were performed at voltage amplitudes up to 5.7 kV and at a frequency of 5.5 kHz. In order to measure the discharge power a small capacitor (*C* = 10 nF) is placed in series between the lower electrode and ground. The voltages applied to the powered and grounded electrode, respectively, are measured with a high voltage probe and serve as *x* and *y* input of a digital oscilloscope. The discharge power is calculated from the resulting Lissajous figures using the standard method based on the area enclosed by the curve and was kept constant (4 W) during the experiments.

### 2.1. Fourier Transform Infrared Reflection Absorption Spectroscopy (FT-IRRAS)

The functional groups present in the films deposited on aluminum coated glass substrates were evaluated by Fourier transform infrared reflection absorption spectroscopy (BRUKER, VERTEX 80V, Karlsruhe, Germany). Prior to the experiments, the sample compartment was evacuated to 2 mbar to reduce absorption of water vapor and CO_2_. The spectra were recorded in the range from 4000 to 500 cm^−1^ with a spectral resolution of 0.7 cm^−1^. Each spectrum was the average of 32 scans. A background spectrum was taken from each substrate prior to the deposition of a-C:H films.

### 2.2. Raman Spectroscopy

Raman spectra for the films were measured with a Raman microscope (Olympus BX 41 linked to a DILOR XY-800 from HORIBA Jobin-Yvon, Bensheim, Germany) using the second harmonics of a Nd:YAG laser (532 nm) as a light source and a CCD-camera (Synapse from HORIBA Jobin-Yvon). The samples were placed on a turntable under the microscope to provide a large sampling area for suitable averaging as well as heat dissipation. To decrease further the local radiation intensity, the measurements were done out of focus at a sample distance of 125 µm using an objective with 50-fold magnification. The resulting laser spot was about 50 µm in diameter. The power of the laser beam was adjusted in the range of 5–20 mW in order to avoid sample damage as well as fluorescence.

### 2.3. Spectroscopic Ellipsometry (SE)

Spectroscopic ellipsometry investigations were carried out with a phase modulated ellipsometer Jobin-Yvon UVISEL (HORIBA Jobin-Yvon). The investigated photon energy region was 1.5–4.5 eV with an energy step of 0.05 eV. The experiments were carried out at room temperature using an angle of incidence fixed at 70° and the compensator was set to 45°. The incidence angle of 70° resulted from Brewster’s angle of the Si (100) wafer substrate. DeltaPsi software (v. 2.6, HORIBA Jobin-Yvon, Bensheim, Germany) was employed to determine the spectral distributions of the refractive index *n*(λ) and the extinction coefficient *k*(λ) of amorphous carbon films.

## 3. Results and Discussion

**Figure 1:**
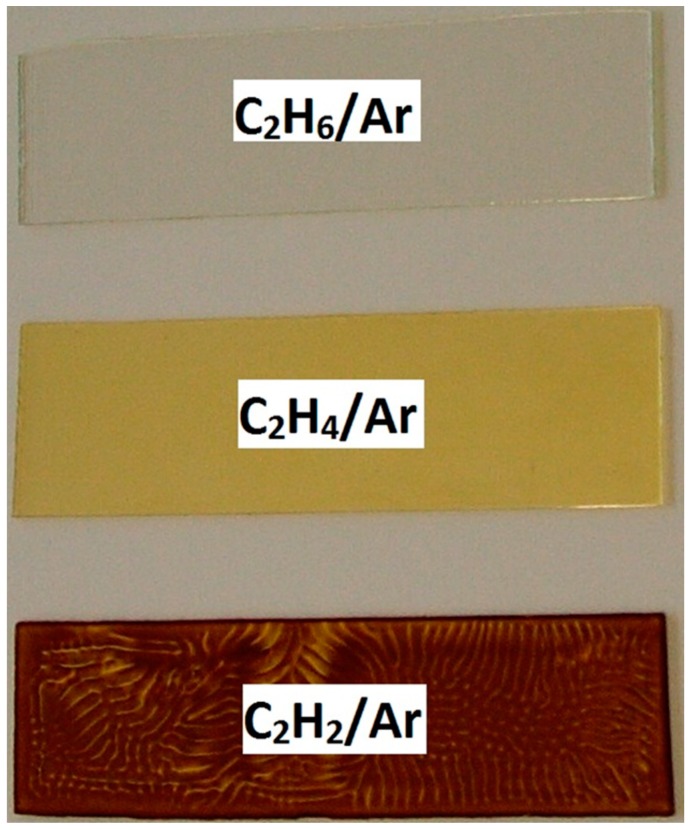
Photographs of a-C:H films deposited on glass substrates in a dielectric barrier discharge (DBD) with different precursor gases C_2_H*_m_*/Ar (*m* = 2, 4, 6).

The physical and chemical properties of a-C:H films obtained from different C_2_H*_m_*/Ar (*m* = 2, 4, 6) gas mixtures are discussed in this section. A photograph displaying the deposited a-C:H films on a glass substrate for different precursor gases is presented in Figure 1. Films deposited with different precursor gases show different colors. Deposited thin films show transparent, yellow, and brown color for C_2_H_6_/Ar, C_2_H_4_/Ar and C_2_H_2_/Ar gas mixtures, respectively. C_2_H_4_/Ar and C_2_H_6_/Ar films were sticky in nature; the C_2_H_2_/Ar film had small particles (of μm size) on its surface. The thickness of the film was in the order of several micrometers after 5 h of deposition.

The deposition rates were estimated from the additional mass deposited on the glass substrate (7.4 × 2.2 cm^2^) which were weighted on a micro balance before and after deposition. Typical deposition rates were in the range 0.08–0.3 µg/(s·cm^2^) (Table 1).

The coloring effect (Figure 1) is the results of optical absorption introduced by *sp* and *sp*^2^ hybridized phases in agreement with our FTIR results, see below. The unsaturated hydrocarbon precursors provide a high level of unsaturated bonding structures of the deposited films (see below). As the amount of *sp* and *sp*^2^ bonded carbon increases, the bonding structure changes. The density of π electrons increases and thus the absorbance of films in the visible range increases. That fact is also proved by an increase of *I*_D_/*I*_G_ ratio as revealed by Raman spectroscopy. Moreover, the thickness of the C_2_H_4_/Ar sample estimated by SE is about two times higher than for C_2_H_6_/Ar. This fact enhances coloring effects as it causes a higher absorbance. Similar brown coloring effects have been observed and reported by Shirakura et al. of diamond-like carbon thin films deposited at PET bottles [27].

The investigated hydrocarbons are characterized by different hydrogen contents and, in the case of acetylene, by a C≡C triple bond. The degree of cross-linking, the hydrogen content, and the fraction of *sp*^2^ and *sp*^3^ bonds are also determined by the plasma conditions, for example, power density and the local ion bombardment during film growth. In generally, one can distinguish between a power-deficit regime when sufficient precursor molecules are present and a monomer-deficit regime at a large discharge power when precursor molecules are completely consumed in the deposition process. Film deposition with C_2_H_6_, C_2_H_4_, and C_2_H_2_ precursors with C-to-H ratios of 0.33, 0.5, and 1 results in cross-linked a-C:H films which show an increasing absorption in the visible and ultraviolet range as is expected from the raising carbon (lower hydrogen) content in the precursor.

### 3.1. Fourier Transform Infrared Reflection Absorption Spectroscopy (FT-IRRAS)

**Figure 2:**
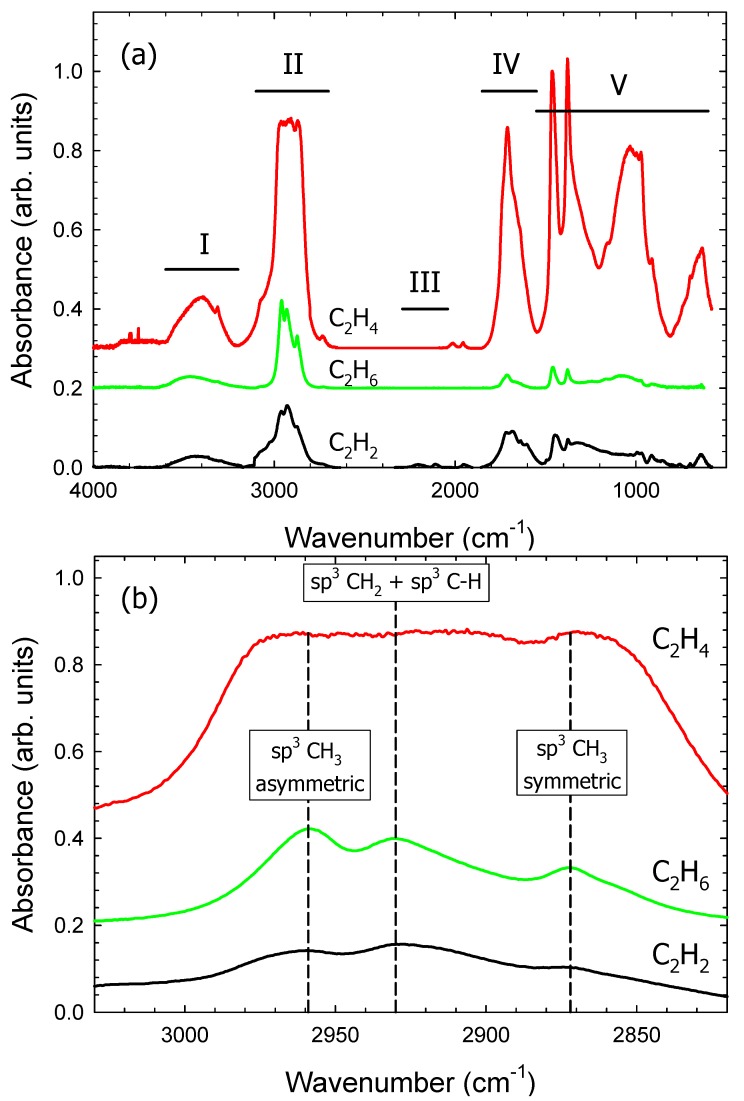
(**a**) FTIR spectra of a-C:H films obtained from C_2_H*_m_*/Ar (*m* = 2, 4, 6) gas mixture, (gas pressure = 300 mbar, gas ratio = 1:2, power = 4 W). To ease comparison, the data obtained with C_2_H_6_/Ar and C_2_H_4_/Ar are shifted upwards by 0.2 and 0.3, respectively; (**b**) Magnification of the FTIR region 3030–2820 cm^−1^. For comparison, the data obtained with C_2_H_6_/Ar and C_2_H_4_/Ar are shifted upwards.

FTIR spectra of a-C:H films deposited on aluminum-coated glass substrates using C_2_H*_m_*/Ar (*m* = 2, 4, 6) gas mixtures at 300 mbar gas pressure with a gas ratio of 1:2 (Figure 2) were measured. The measured spectra can be subdivided into five spectral regions I, II, III, IV, and V corresponding to the intervals 3200–3600 cm^−1^, 2700–3100 cm^−1^, 2040–2290 cm^−1^, 1550–1850 cm^−1^, and 600–1550 cm^−1^, respectively. In general, the C_2_H_4_/Ar and C_2_H_6_/Ar films display a rather similar absorbance while significant differences compared to the C_2_H_2_/Ar film are noticeable.

Region I (3200–3600 cm^−1^): The broad band arises due to hydroxyl groups. There are two reasons for the presence of oxygen and hydroxyl groups. The first is associated with atmospheric moisture [28] which is an impurity caused during film transfer from DBD chamber to FTIR chamber. We tried to avoid this by quickly moving the sample to the FTIR chamber. Reducing the impurity level to zero in this process was highly impossible, however. The second reason which comes to mind is the surface of electrodes which by itself contain oxygen which could be released through chemical reactions during plasma operation. Other functional groups associated with oxygen are observed in regions IV and V.

Region II (2700–3100 cm^−1^) shows the strongest absorbance; it is related to *sp*^2^ and *sp*^3^ CH_x_ stretching vibrations [26,29,30,31]. Polymer-like films exhibit about 40%–60% of *sp*^2^ and *sp*^3^ hybridized C–H bonds in their structure. The magnification of the region from 2700 to 3100 cm^−1^ (Figure 2b) shows the presence of different modes of C–H vibrations in all three films. The bands at 2872 cm^−1^, 2930 cm^−1^ and 2959 cm^−1^ are related to *sp*^3^ CH_3_ symmetric, superimposing of *sp*^3^ CH_2_ asymmetric and *sp*^3^ C–H mode, and *sp*^3^ CH_3_ asymmetric C–H vibrations, respectively [26,29,30,31]. The relative absorbance of these bands is much higher than for DLC films [31,32,33,34]. The C_2_H_6_/Ar film shows the strongest absorption near 2900 cm^−1^ and consequently the highest *sp*^3^ CH_3_ configuration compared to the other two films. All these bands are characteristic of a hydrogen-rich carbon film. From Table 2, the largest FTIR absorbance can be found in this region for all films.

Region III (2040–2290 cm^−1^) provides the absorbance of chemical bonds containing C≡C functional groups. Thin films deposited from C_2_H_4_/Ar and C_2_H_6_/Ar gas mixtures do not show any absorption in this region. The C_2_H_2_/Ar film shows a weak doublet at 2200 and 2100 cm^−1^ which is related to stretching of C≡C bonds. Thin films deposited under the same experimental parameters from C_2_H_2_/N_2_, C_2_H_4_/N_2_ and C_2_H_6_/N_2_ gas mixtures shows absorbance in this region which, however, is largely due to nitrile and isonitrile groups [35].

Region IV (1550–1850 cm^−1^) is associated with C=C and C=O stretching vibrations [26,30] and C–H bending vibrations. The broad band at 1700 cm^−1^ is related to C=C and C=O stretching vibrations. The intensity of this band is smallest for the C_2_H_6_/Ar film.

Region V: Bands at 1459 cm^−1^ and 1370 cm^−1^ are related to C–H_x_ bending vibrations [26,30]. The broad band at 1024 cm^−1^ is due to C–O stretching and O–H deformation vibrations. The band at 640 cm^−1^ is from the benzene ring which is related to out-of-plane C–H deformations. All three films show the same functional groups but with different absorbances.

The relative absorbance of the five regions (with respect to the integral absorbance of the measured spectral range) is shown in Table 2. Distinct differences are noted. For example, region II (2700–3100 cm^−1^) shows a pronounced absorbance in all three spectra; the region dominates in the films deposited with C_2_H_2_ and C_2_H_6_ precursors and is equally strong as region V (1200–1550 cm^−1^) of the film deposited with C_2_H_4_. The strong absorbance in this region confirms the films contain a significant amount of hydrogen. Region III is the weakest of all regions and only absorbing in the film from the C_2_H_2_ precursor. Region IV is fairly strong in the C_2_H_2_ and C_2_H_4_ films but much weaker in the C_2_H_6_ film.

A quantitative analysis of the molecular structure by infrared (FTIR) and Raman spectroscopy is difficult to perform and was not attempted here. In particular, due to different and largely unknown cross sections the peak absorbance is not a measure for the content of specific molecular groups. The absorbance is additionally influenced by the non-uniform molecular surrounding due to crosslinking and interactions between molecular groups. As a consequence, the center peak intensity decreases and the absorption bands become broadened.

### 3.2. Raman Spectroscopy

**Figure 3:**
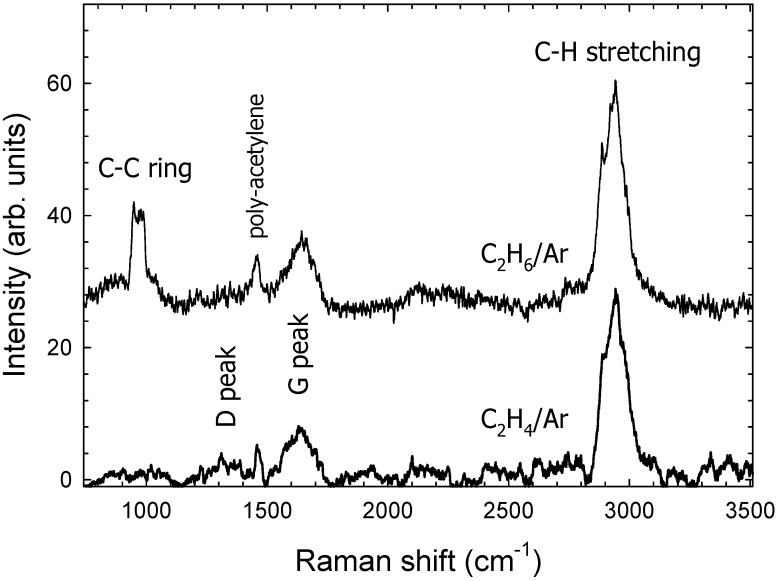
Raman spectra of a-C:H films obtained from C_2_H*_m_*/Ar (*m* = 4, 6) gas mixtures, (gas pressure *p* = 300 mbar, gas ratio 1:2, power *p* = 4 W).

Raman spectra for C_2_H_4_/Ar and C_2_H_6_/Ar films are displayed in Figure 3. Raman spectroscopy is a standard nondestructive tool for the characterization of crystalline, non-crystalline, and amorphous carbon. Raman spectra for C_2_H_4_/Ar and C_2_H_6_/Ar films show similar characteristics. Generally, Raman spectra of disordered graphite show two sharp modes, so-called G and D peaks which we observe around 1640 cm^−1^ (G-peak) and 1355 cm^−1^ (D-peak); the G-peak (*I*_G_) is frequently assigned to the E_2g_ symmetric vibration mode of graphite layers in the film [36,37]. The D and G peaks are both related to *sp*^2^ bonds. The G peak comes from the stretching vibrations of *sp*^2^ bonds either from C=C chains or aromatic rings while the D peak corresponds to the breathing mode of *sp*^2^ bonds only in rings [28]. The evaluated *I*_D_/*I*_G_ intensity ratio was about 0.45 and 0.3 for the C_2_H_4_/Ar and the C_2_H_6_/Ar film, respectively. The small *I*_D_/*I*_G_ intensity ratio shows that C_2_H_4_/Ar and C_2_H_6_/Ar films contain a significant amount of hydrogen and have a large fraction of *sp*^2^ hybridized bonding. In addition we observe a sharp peak at 1460 cm^−1^ which is assigned to the to a C5 Ag_(2)_ pentagonal pinch mode in trans-polyacetylene [6,38]. Polyacetylene consists of long chains of CH groups with alternating single and double bonds between the carbon atoms. The amount of polyacetylene is believed to be small, however, as it has a large Raman cross section [6].

Almost identical I_D_/I_G_ ratios have been observed by Olivera et al. [39] for films that were prepared using PECVD. The band at 2937 cm^−1^ is related to C–H stretching vibrations which are also observed with FTIR spectroscopy. No Raman measurements were carried out for the C_2_H_2_/Ar film due to poor film quality. We do expect a larger *I*_D_/*I*_G_ ratio caused by a smaller hydrogen content for the C_2_H_2_/Ar film [6,38].

### 3.3. Ellipsometry

In this study, fitting calculations were based on a four-phase optical model (ambient/amorphous carbon/SiO_2_/Si-wafer). Amorphous carbon was assumed as an isotropic, homogeneous material and its dispersion was simulated by the Tauc-Lorentz oscillator (TL) model. We used a recently proposed dispersion model for amorphous semiconductors by Gioti et al. and Logothetidis et al. [40,41]. The TL model is a wide-spread used approach for the description of dispersion of the optical properties of a-C, a-C:H and layers deposited by DBD discharge. This model is the combination of the Tauc joint density of states [40] and the quantum mechanical Lorentz oscillator model [42]. TL model fits to the dielectric functions of amorphous material class, which exhibit a peculiarity due to the presence of two separated contribution of inter-band electronic transition related to *sp*^2^ and *sp*^3^ bonded carbon [43]. The appropriate parameters of TL model were applied for the analyzed layer considering different film structure which was derived from Raman results.

**Figure 4:**
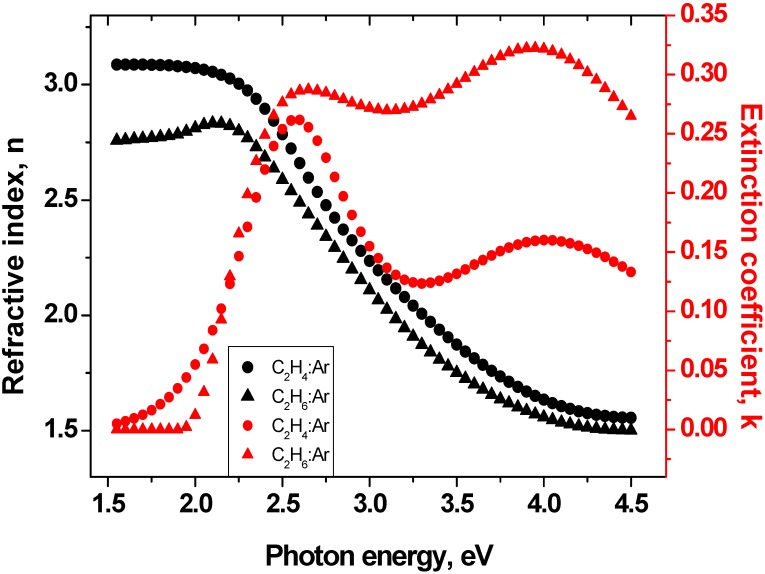
Wavelength-dependence of the optical constants *n*(λ) and *k*(λ) for the films prepared with C_2_H_4_/Ar and C_2_H_6_/Ar gas mixtures.

Moreover, the dispersion of Si (100) wafer covered by a 2.2 nm thin SiO_2_ native oxide films were taken from database [44]. The assumed optical model was fitted to the experimental data by a non-linear Levenberg-Marquardt regression [45] using mean-square error minimization. The fitting procedure described above gives accurate values of film thicknesses as well as variation of the refractive index and extinction coefficient versus wavelength. Spectral variations of *n*(λ) and *k*(λ) of samples are presented in Figure 4. *n*(λ) and *k*(λ) appear like quantities connected by the Kramers–Kronig relations [45]. This means that *n*(λ) shows an inflection point at a wavelength where *k*(λ) exhibits maximum values in the visible wavelength regime (at about 2.6 eV or 477 nm). Refractive indices decrease from low to high energies. They correspond to 2.81 and 3.08 at 2 eV (633 nm), respectively, for C_2_H_6_/Ar and C_2_H_4_/Ar samples.

These values are reasonable higher than optical constants of a-C:H thin rf PE CVD films (*n* ≈ 1.7–2.2) grown in a CH_4_/H_2_ gas mixture [46,47] or a-C:H thin dc PE CVD films (*n* ≈ 2.2) deposited using acetylene precursors [48]. It is worth noting that the rf sputtered a-C films studied by Gioti et al. [42] also show refractive indices of 2.2–3.0 in the visible wavelength range. A comparable refractive index (*n* ≈ 2.6–2.8) was obtained for hydrogenated carbon films deposited in an Ar/H_2_ gas mixture using an expanding thermal plasma CVD process [49] or a filtered cathodic vacuum arc (FCVA) [50].

**Figure 5:**
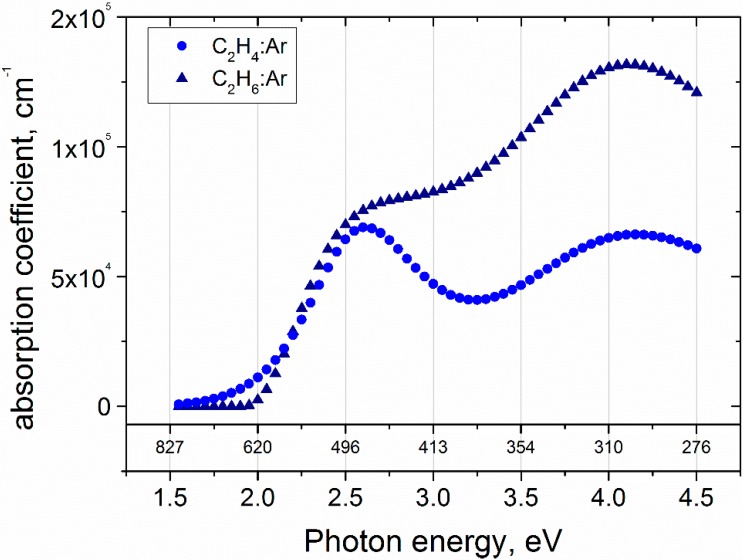
Absorption coefficients for the films prepared with C_2_H_4_/Ar and C_2_H_6_/Ar gas mixtures obtained from spectroscopic ellipsometry.

The maximum extinction coefficient of the C_2_H_6_/Ar sample (0.32) occurs at 4 eV. It comes from electronic transitions and causes absorption in the UV. It is higher than the highest *k*(λ) value of the C_2_H_4_/Ar sample (0.27) because this sample contains more *sp*^2^ hybridized phase. Deposition using C_2_H_6_ decreases the amount of *sp*^3^ hybridized phase. It could be also observed as a lower *I*_D_/*I*_G_ ratio extracted from Raman spectroscopy results. Generally, the spectral variation of the extinction coefficient exhibits smaller values for C_2_H_4_/Ar samples compared to C_2_H_6_/Ar films. This yields values close to zero below 2 eV photon energy, which is indicated as a high film transparency in the visible wavelength regime and is typical for DLC and a-C:H films (Figure 5). The optical band gap, calculated by the Tauc relationship [51], exhibits slightly higher values for C_2_H_6_/Ar sample (see Table 3). However, the obtained *E*_og_ values are typical for carbon containing *sp*^3^ hybridized fractions.

The thickness of the C_2_H_4_/Ar sample is about two times higher than for C_2_H_6_/Ar. Moreover, this difference also comes from lower activity of the C_2_H_6_ precursor in layer nucleation concerned with higher level of saturation of the carbon bonds in this hydrocarbon. The type of hydrocarbon also influences carbon incorporation into the layer and types of bonding hybridization. Ellipsometry measurement for C_2_H_2_/Ar films was not successful since the deposited film contained too many small micro-particles on its surface.

A systematic investigation of hydrogenated carbon films deposited with CH_4_ and C_2_H_2_ gas mixtures and employing different plasma deposition techniques has been carried out by Casiraghi et al. [6]. According to these studies, the optical band gap increases with increasing hydrogen content while at the same time the *I*_D_/*I*_G_ ratio decreases. Comparing our data with their results we can infer that the hydrogen contents of the present C_2_H_4_/Ar and C_2_H_6_/Ar films should be around 26% and 31%, respectively. If correct, these values are close to the H content of hydrogenated tetrahedral carbon films which are intermediate between DLC and polymer-like films [6].

## 4. Conclusions

Amorphous hydrogenated carbon films were successfully deposited on Si (100) and aluminum coated glass substrates using a dielectric barrier discharge with C_2_H*_m_*/Ar (*m* = 2, 4, 6) gas mixtures at a pressure of 300 mbar. Films show attractive and different yellow/brown colors which could be further tailored for use as decorative coatings. FT-IRRAS shows strong absorptions near 2900 cm^−1^ and at 1459 cm^−1^ and 1370 cm^−1^ which are related to CH_2_ and CH_3_ stretching and bending vibrations, respectively, of the *sp*^3^ configuration. Some differences between the different gas mixtures are noted. The C_2_H_6_/Ar film shows the strongest absorption near 2900 cm^−1^ and consequently the highest *sp*^3^ CH_3_ bonding compared to the other two films. C≡C functional groups are weak and only observed for the film from the C_2_H_2_/Ar gas mixture. The presence of carboxyl groups is an indication that some post-deposition oxidation has occurred. Raman spectroscopy reveals the presence of D and G bands at 1643 and 1350 cm^−1^, respectively. The measured I_D_/I_G_ intensity ratio is 0.45 and 0.3 for the C_2_H_4_/Ar and C_2_H_6_/Ar films, respectively. An additional peak observed at 1460 cm^−1^ is assigned to the pentagonal pinch mode in trans-polyacetylene. Spectroscopic ellipsometry reveals that thin films obtained from C_2_H_6_/Ar and C_2_H_4_/Ar gas mixtures are characterized by a high refractive index of 2.81 and 3.07, respectively, at a photon energy of 2 eV. The extracted optical band gap is 1.91 eV and 1.75 eV for the C_2_H_6_/Ar and C_2_H_4_/Ar films. The observations are consistent with a significant hydrogen content of about 26% and 31% in the C_2_H_4_/Ar and C_2_H_6_/Ar films.

## Figures and Tables

**Table 1:** Mean deposition rates for different precursor molecules.

Gas Mixture	Deposition Rate in 10^−5^ kg/(s·m^2^)
C_2_H_6_/Ar	0.08
C_2_H_4_/Ar	0.18
C_2_H_2_/Ar	0.28

**Table 2:** Relative absorbance of regions I, II, III, IV, and V (with respect to the integral absorbance of the measured spectral ranges).

Region	Wavenumber (cm^−1^)	Relative Absorbance		
		C_2_H_2_	C_2_H_4_	C_2_H_6_
I	3200–3600	0.11	0.09	0.19
II	2700–3100	0.40	0.35	0.58
III	2040–2290	0.01	0.00	0.00
IV	1550–1850	0.22	0.20	0.07
V	1200–1550	0.26	0.36	0.15

**Table 3:** Parameters of amorphous carbon samples obtained from spectroscopic ellipsometry and Raman spectroscopy.

Sample	*n* (@ 2 eV)	*E*_og_ (eV)	*I*_D_/*I*_G_ Ratio
C_2_H_4_/Ar	3.07	1.75	0.45
C_2_H_6_/Ar	2.81	1.91	0.3
